# The modification role and tumor association with a methyltransferase: KMT2C

**DOI:** 10.3389/fimmu.2024.1444923

**Published:** 2024-08-06

**Authors:** Yunjuan Jiao, Yuanhao Lv, Mingjie Liu, Yun Liu, Miaomiao Han, Xiwen Xiong, Hongyan Zhou, Jiateng Zhong, Xiaohong Kang, Wei Su

**Affiliations:** ^1^ Department of Pathology, The First Affiliated Hospital of Xinxiang Medical University, Xinxiang, China; ^2^ Department of Pathology, Xinxiang Medical University, Xinxiang, China; ^3^ Henan Health Commission Key Laboratory of Gastrointestinal Cancer Prevention and Treatment, The First Affiliated Hospital of Xinxiang Medical University, Xinxiang, China; ^4^ Xinxiang Key Laboratory of Precision Diagnosis and Treatment for Colorectal Cancer, Xinxiang First People’s Hospital, Xinxiang, China; ^5^ Xinxiang Engineering Technology Research Center of Digestive Tumor Molecular Diagnosis, The First Affiliated Hospital of Xinxiang Medical University, Xinxiang, China; ^6^ Department of Oncology, The First Affiliated Hospital of Xinxiang Medical University, Xinxiang, China

**Keywords:** histone methylation, KMT2C, biological process, cancer, immunotherapy

## Abstract

Histone methylation can affect chromosome structure and binding to other proteins, depending on the type of amino acid being modified and the number of methyl groups added, this modification may promote transcription of genes (H3K4me2, H3K4me3, and H3K79me3) or reduce transcription of genes (H3K9me2, H3K9me3, H3K27me2, H3K27me3, and H4K20me3). In addition, advances in tumor immunotherapy have shown that histone methylation as a type of protein post-translational modification is also involved in the proliferation, activation and metabolic reprogramming of immune cells in the tumor microenvironment. These post-translational modifications of proteins play a crucial role in regulating immune escape from tumors and immunotherapy. Lysine methyltransferases are important components of the post-translational histone methylation modification pathway. Lysine methyltransferase 2C (KMT2C), also known as MLL3, is a member of the lysine methyltransferase family, which mediates the methylation modification of histone 3 lysine 4 (H3K4), participates in the methylation of many histone proteins, and regulates a number of signaling pathways such as EMT, p53, Myc, DNA damage repair and other pathways. Studies of KMT2C have found that it is aberrantly expressed in many diseases, mainly tumors and hematological disorders. It can also inhibit the onset and progression of these diseases. Therefore, KMT2C may serve as a promising target for tumor immunotherapy for certain diseases. Here, we provide an overview of the structure of KMT2C, disease mechanisms, and diseases associated with KMT2C, and discuss related challenges.

## Introduction

Post-translational modification (PTM) of proteins is the covalent modification of amino acid side chains in translated proteins. It can expand the functional diversity of proteins by regulating protein folding, activity, stability, localization, signal transduction and binding under physiological and pathological conditions (1). Its main forms include ubiquitination, phosphorylation, methylation, acetylation, glycosylation and succinylation (2). It is closely related to immune cell activation, signal regulation, immune response and tumor metabolic reprogramming (3–5). It can directly or indirectly affect the efficacy of immunotherapy by modulating immune checkpoints or remodeling the tumor immune microenvironment (6–8). Numerous studies have demonstrated that aberrant post-translational modifications of proteins can affect cancer development by regulating tumor metabolic reprogramming (9). Histone modification is the process by which histones are methylated, acetylated, phosphorylated, adenylated, ubiquitinated, ADP-ribosylated, etc. by the action of relevant enzymes. Histone methylation occurs predominantly on lysine or arginine residues in H3 and H4 and regulates cellular metabolic processes by activating or repressing gene expression (10, 11).

Mutations and translocations of histone lysine methyltransferases (KMTs) and lysine demethylases (KDMs) are common mechanisms driving tumorigenesis (12–17). Thus, both KMTs and KDMs are potential therapeutic targets for human cancer (18–21). KMTs were classified into six subfamilies based on major amino acid sequences and substrate specificity (22). SET1A, SET1B and MLL1-4 belong to the KMT2 family and catalyze mono-, di- and trimethylation of histone H3 lysine position 4, which is proposed to be involved in the positive regulation of gene transcription (23, 24). This family of enzymes was originally isolated biochemically from yeast as a macromolecular complex named COMPASS (Complex Proteins Associated with Set1) (24, 25).

Histone-lysine N-methyltransferase 2 (KMT2) family genes, also known as MLL genes, are frequently mutated in various types of cancer (26). The KMT2 complex methylates histone 3 lysine 4 (H3K4) to regulate DNA accessibility and transcription. Unlike other COMPASS family members that trimethylated H3K4, MLL3 (KMT2C) and MLL4 catalyze histone H3K4 monomethylation at the enhancer (27, 28) and are the most common mutant histone modifiers in human cancer (29). KMT2C mutations have been detected in a variety of tumors, including hepatocellular carcinoma (30), breast cancer (31), colon cancer (32), bladder cancer (33), myelodysplastic syndromes and acute myeloid leukemia (AML) (34). In a PI3K-driven breast tumor model, KMT2C interacts with the FOXA1 transcription factor and mutational inactivation leads to increased mammary stem cell activity and accelerated tumor progression (35, 36). In addition, chromosome fragmentation leading to rearrangement of KMT2C has been reported in colon cancer (37). In this review, we will focus on KMT2C, the major mammalian histone methyltransferase. Its established importance in gene regulation and mutation frequency in developmental diseases and cancers warrants an exploration of the literature to stimulate further research and development of new therapeutic approaches. Thus, we focus on the structure of KMT2C as well as the cancers associated with KMT2C and its mechanism of action in cancer, and discuss the related challenges in the hope of providing possible application value.

## The structure of KMT2C

**Figure 1:**
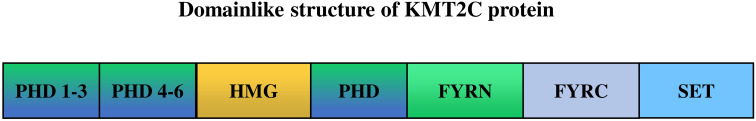
The structure of KMT2C (UniProt ID: Q8NEZ4). PHD, the plant homeodomain (PHD) fingers; HMG, high mobility group domains; FYRN, “FY-rich” domain, N-terminal region; FYRC, “FY-rich” domain, C-terminal region; SET, SET(Su(var)3-9, Enhancer of zeste, Trithorax) domain. KMT2C PHD fingers 1–3 act as “readers” of the histone methylation status, recognizing monomethylated H3K4 (H3K4me1), while the SET domain, located in the Cterminus, is the “writer” that adds methyl- groups to complete the methylation process.

KMT2C, also known as MLL3, localized to chromosome 7q36.1, is a member of the TRX/MLL gene family and is a histone methyltransferase that specifically catalyzes the monomethylation of histone H3 lysine K4 in enhancer regions (38, 39), thought to be involved in tissue growth regulation, tumorigenesis and transcriptional co-activation (40). As shown in **Figure 1**, the gene encodes 4911 amino acids and contains seven plant homologous structural domains (PHD), a high mobility group structural domain, two FY (phenylalanine tyrosine) enriched regions, and a SET (Su(var)3-9, Enhancer-of-zeste,Trithorax) structural domain. PHD and SET structural domain proteins are chromatin regulators, some of which are altered in cancer (41). Researchers find KMT2C PHD mutations can expose vulnerability to EZH2 inhibitor therapy by deregulating tumor suppressors (42).

The KMT2C protein binds and methylates the enhancer region of histone 3 lysine 4 (H3K4), promoting the recruitment of transcriptional activators involved in DNA replication restarting (43, 44). Studies have shown that KMT2C has an oncostatic effect and is frequently found in solid tumors inactivated by deletion or mutation of this gene. Deletion of the catalytic core of the SET structural domain in KMT2C induces cell hyperproliferation and uroepithelial tumor formation (45), but knockout of the entire gene is lethal in mice (46). Recently, several studies on polymorphisms in the KMT2C gene have found positive associations with colorectal and pancreatic cancers (47, 48). Furthermore, the KMT2C gene is associated with TKI resistance and may be a potential biomarker for predicting the prognosis of cancer development (49, 50).

## KMT2C regulates cellular biobehavioral functions in cancer development

**Figure 2:**
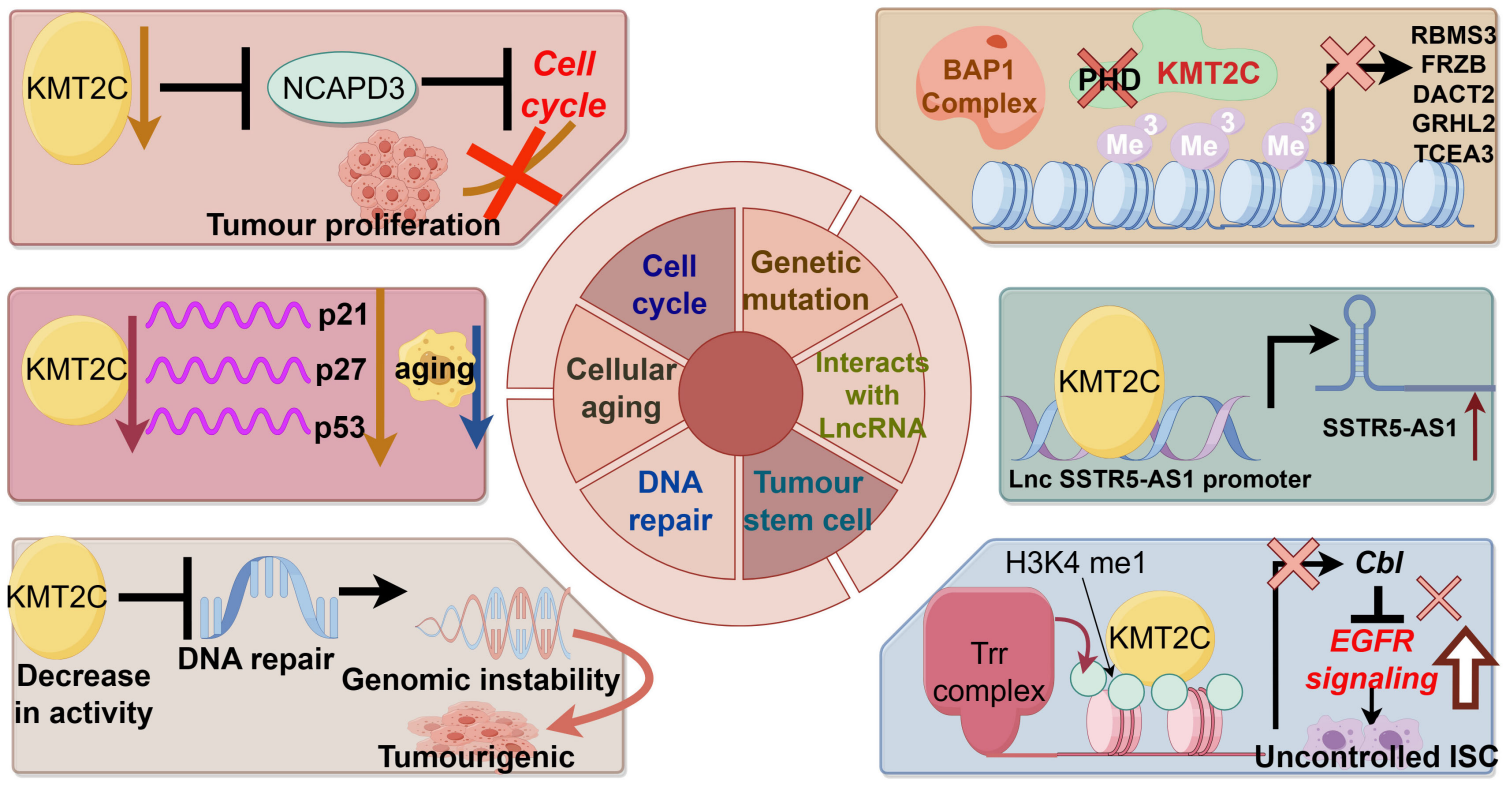
The biological function of KMT2C in promoting cancer development.

**Figure 2** demonstrates the biological function of KMT2C in promoting cancer development.

### Genetic mutation

The MLL family is known as myeloid/lymphoid or mixed-spectrum leukemia proteins, and mutations, deletions and low expression of the gene KMT2C (MLL3) appear to play an important role in the development of leukemia. Chromosomal translocations are the most common form of MLL mutation. Chromosomal translocation fuses the MLL gene with a chaperone gene to form a new fusion protein that promotes leukemia (51). Other studies suggest that loss of KMT2C may contribute to the development of myelodysplastic syndromes and acute myelocytic leukemia (AML) by promoting myelopoiesis (52). In addition, the reduction of KMT2C gene expression can synergize with other factors at chromosome 7q to promote AML (34). Abnormalities in DNA methylation caused by germline mutations in the KMT2C gene were found in Chinese AML and colorectal cancer families and may be responsible for the pathogenesis of patients in the family lines (32, 53). Recent second-generation sequencing has also identified a heterozygous deletion due to a nonsense mutation in KMT2C that promotes human T-cell virus-induced acute T-cell leukemia (54).

### Interaction with long non-coding RNA and RNA interacting with PIWI protein

Non-coding RNAs can interact with KMT2C to regulate gene expression. Wang et al. found that LncRNA SSTR5-AS1 increased the enrichment of KMT2C and H3K4me3 in the promoter region of the growth inhibitory receptor 5 by interacting with KMT2C and induced the transcription of the oncogene growth inhibitory receptor 5 in laryngeal squamous carcinoma (55). In addition, the LncRNA HOTAIR, which is highly expressed in tumors such as breast cancer, can mediate oncogene silencing by interacting with KMT2C, and its mechanism of action is also related to promoter activity (56). He et al. also found that piRNA effectively up-regulated the transcription of apoptosis-inducing ligands related to the pro-apoptotic protein tumor necrosis factor (TNF) by inducing the methylation of H3K4/demethylation of H3K27, thereby inhibiting tumor growth (57).

### Cellular aging

The absence of cellular senescence mechanisms has been suggested as a possible reason for the unlimited proliferation of tumor cells. Xia et al. found that KMT2C promoted senescence of esophageal squamous carcinoma cells, and its mRNA level was down-regulated in esophageal squamous carcinoma tissues. Knockdown of KMT2C down-regulated senescence factors p21, p27 and p53 mRNA levels, while KMT2C overexpression up-regulated senescence factor levels (58). The mutation rate of KMT2C was found to be higher in breast cancer patients >50 years of age than in those <50 years of age, which may reflect the correlation between KMT2C mutations and cellular senescence (59).

### Cell cycle

Disturbance of the cell cycle is an important mechanism of tumorigenesis, and abnormalities of various molecules that regulate the cell cycle can cause tumorigenesis. Dawkins et al. experimented with eight human pancreatic cell lines and showed that cell proliferation was reduced in the presence of methyltransferase depletion, possibly due to cell cycle arrest. Enrichment analyses of the gene sets following KMT2C and KMT2D knockdown showed significant down-regulation of genes related to cell cycle and growth (60). Yuan et al. found that the proliferation of human hepatocellular carcinoma cells HepG2 was significantly inhibited after the expression of KMT2C was down-regulated by the interference of small interfering RNAs, and flow cytometry showed that the cells were mainly blocked in the S phase. KMT2C is an H3K4 monomethyltransferase, and silencing of this gene blocks the normal modification of histone proteins, which may result in cell cycle arrest in S-phase, thus inhibiting cell proliferation.

### DNA repair

DNA repair is a cellular reaction in which damaged DNA molecules are restored to their normal DNA sequence structure and genetic information is maintained relatively stable by the action of various enzymes. Abnormalities in the DNA repair system can lead to tumor development. Bladder cancer cells with low KMT2C activity lack homologous recombination-mediated DNA repair of double-strand breaks, resulting in greater endogenous DNA damage, leading to genomic instability and promoting tumorigenesis (61). Another experiment showed that, in order to resist Adriamycin-induced DNA damage, MLL3/4 collaborated with the ASCOM complex to increase the methylation level of H3K4, activate p53, increase the expression of endogenous p53 target genes, and participate in the tumor-suppressing pathway of p53 (45).

### Tumor stem cells

Tumor stem cells are important for tumor survival, proliferation, metastasis and recurrence. Tumor stem cells maintain the viability of the tumor cell population through self-renewal and unlimited proliferation. KMT2C-related H3K4 methylation is thought to be associated with stem cell self-renewal (62). KMT2C also regulates the proliferation and transformation of tumor stem cells, and Gervais found that KMT2C regulates the proliferation of intestinal stem cells, and that its absence leads to an increase in the level of EGFR proteins, which promotes the self-renewal of intestinal stem cells and the overgrowth of tumor-like stem cells (63). Another study found that knockdown of KMT2C in gastric epithelial cells promoted epithelial-to-mesenchymal transition and enhanced the expression of mesenchymal transition-associated proteins, and that migration and invasion of gastric epithelial cells were increased 47-88-fold by knockdown of KMT2C (64).

In conclusion, mutations in the KMT2C gene alter the epigenetic state of chromatin and affect gene transcription. In the case of KMT2C wild type, epigenetic regulation of chromatin is in equilibrium. The KMT2C complex can be recruited to the enhancer by the BAP1 complex to catalyze H3K4me1 and enhance gene transcription. However, in tumors with KMT2C mutations, the KMT2C complex is not recruited to BAP1-dependent enhancers, and the increased level of repressive H3K27me3 leads to an imbalance in the epigenetic state of enhancer chromatin, increased chromosome stability, and reduced transcriptional activity, which may result in silencing of tumor suppressor genes (42).

## The role of KMT2C in cancer

**Figure 3:**
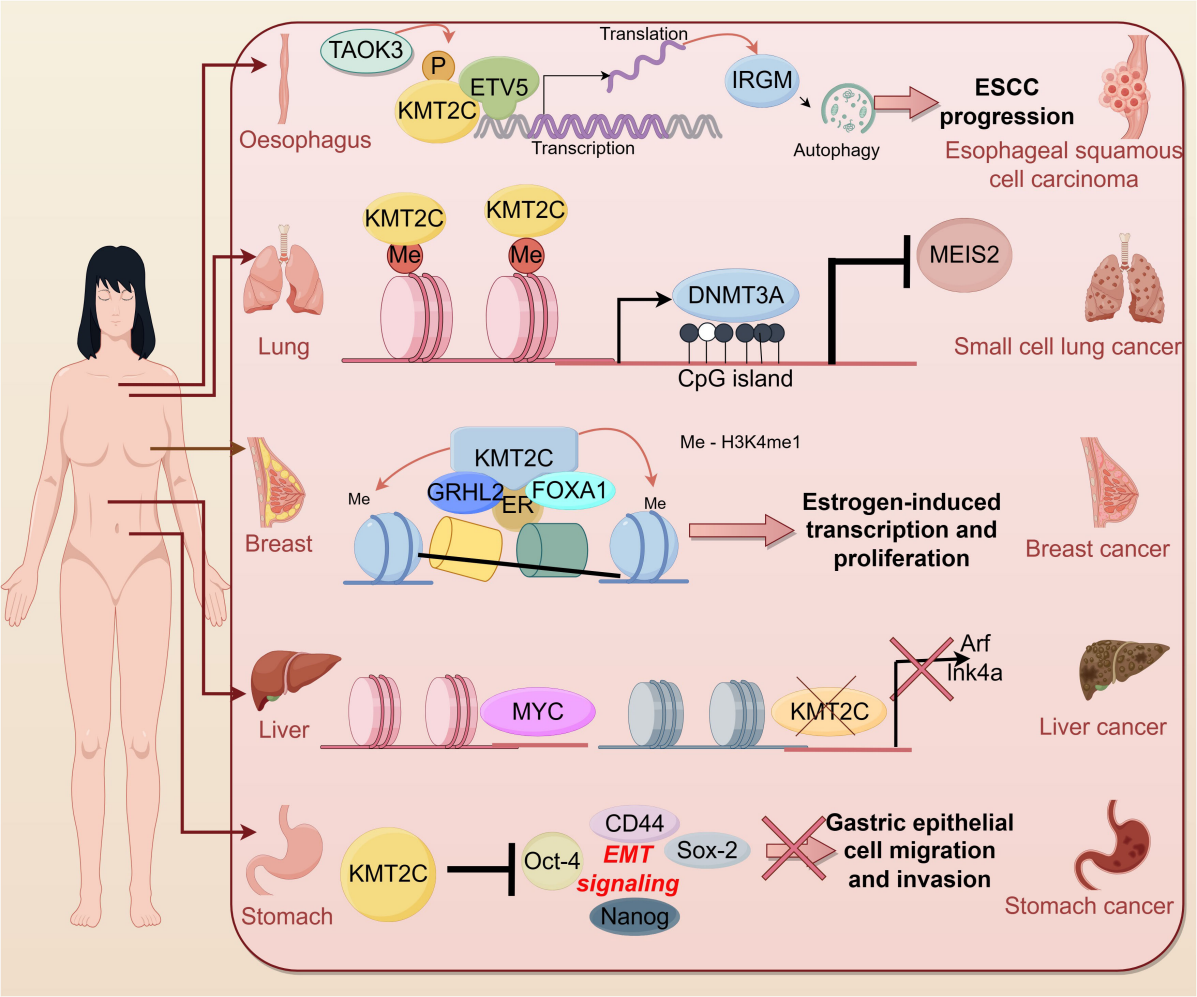
Role of KMT2C in cancer.

In recent years, more and more studies have shown the correlation between KMT2C and tumors. According to cBioportal, KMT2C is frequently mutated in a variety of cancer types such as breast cancer (~ 12%), melanoma (~ 45%), colon cancer (~ 14%) and hepatocellular carcinoma (50%) (30). KMT2C was found to be aberrantly expressed in cell lines and tumor tissues of many tumor patients, suggesting that KMT2C is involved in many cancer-related signaling pathways (**Figure 3**).

### Digestive system cancer

#### Liver cancer

One study identified HMT genes with significant rates of genetic alteration in 360 hepatocellular carcinoma (HCC) samples. Among them, KMT2C had the highest mutation rate in HCC samples at 5.6 (65), and is associated with a poor prognosis (66). In particular, higher KMT2C mutation rates were detected in adolescent and young adult patients with advanced cholangiocarcinoma, which may suggest that cholangiocarcinoma is more aggressive in adolescent and young adult patients (67).

KMT2C, a histone methyltransferase involved in transcriptional co-activation and co-repression, is seen as a silent/intronic mutation or structural alteration in hepatic metastases from breast, melanoma, and colon cancer. Mutations in KMT2C may enhance homing and habituation of hepatocellular carcinoma cells in the liver (68). Thus, common KMT2C mutations/rearrangements may be a driver not only for primary tumors but also for liver metastases. Researchers find KMT2C, which is mutated in >50% of hepatocellular carcinomas, is also mutated in liver metastases (68).

In a recent study, Zhu et al. concluded that KMT2C likely acts as a tumor suppressor to limit Myc-driven hepatocellular carcinomas, identifying multiple tumor suppressor programs regulated by KMT2C including cell-autonomous mechanisms (cellular metabolism) and non-autonomous mechanisms (interactions with the extracellular matrix and the immune system) (69). Mechanistically, the CDKN2A locus is a genomic and transcriptional target of KMT2C in hepatocellular carcinoma cells, and KMT2C mediates oncogene-induced apoptosis in a CDKN2A-dependent manner.

In addition to its role in hepatocellular cancers, KMT2C regulates bile acid (BA) homeostasis by modulating the expression of Farnesoid X Receptor (FXRs) and p53-dependent small heterodimeric chaperone receptors (SHPs) (70).

#### Gastric cancer

Mutations and low expression of the KMT2C gene are associated with a high risk of progression and poor prognosis in gastric cancer patients and are independent predictors of disease recurrence (71–74). In addition, it has been shown that KMT2C has a high mutation rate in different metastatic sites occurring in gastric cancer patients: peritoneal metastasis (42%), haematogenous metastasis (67%), and distant lymph node metastasis (35%) (75). Zhou et al. found that KMT2C mutations were predominantly detected in HER2 + gastric cancer samples (7/10) and were also associated with the lysine degradation pathway (76).

Cho et al. showed that *in vitro* knockdown of KMT2C promotes migration and invasion of gastric epithelial cells by promoting the EMT signaling pathway (64). Specifically, there is an increase in KMT2C mutations, which show higher levels of “clock-like” mutational features, increased genome-wide doubling, chromosomal instability (especially copy number loss), reprogrammed microenvironments, enriched cell-cycle pathways, Myc activation, and impaired immune responses (77).

Evidence that KMT2C mutations are potential predictors of immunotherapy response in solid malignancies (78). However, in contrast to the high mutation rate of KMT2C, which promotes migration and invasion of gastric epithelial cells, the high mutation rate of KMT2C tends to show a better prognosis in patients treated with immune checkpoint inhibitors (ICIs), and the KMT2C mutation is significantly and positively correlated with higher intratumoural infiltration of CD8+ lymphocytes, CD163+ tumor-associated macrophages, and PD-L1 in gastric cancer tumors (79).

#### Pancreas cancer

Cancers with high TMB have been shown to respond better to ICI, such as pancreatic adenocarcinoma (PAAD) (80). PAAD patients with KMT2C mutations have a higher severity of tumor mutational burden (TMB) and a worse prognosis, mainly related to the fact that mutations in the KMT2C gene affect the composition of immune cells in PAAD patients and positively regulate metabolic and protein-related pathways in PAAD (81). These all suggest that KMT2C mutations may serve as biomarkers for predicting the prognosis of PAAD patients and guiding anti-PAAD immunotherapy.

Notably, in a 2016 article, Dawkins et al. suggest that targeting KMT2C in PDAC may also have therapeutic benefit, especially in those patients who exhibit higher KTM2C expression (60). They found by biopsy that low expression of KMT2C also demonstrated a better prognosis, and experiments on eight human pancreatic cell lines showed that when KMT2C was depleted, cell cycle arrest and reduced progression from G0-G1 led to attenuated cell proliferation.

Furthermore, KRAS mutation is a marker for pancreatic ductal adenocarcinoma (PDAC) and is associated with key aspects of its biology, such as inflammation, immune escape and metabolic alterations (82–85). Mutant KRAS is a major oncogenic driver of PDAC and an attractive therapeutic target (86, 87). However, KRAS wild-type (WT) is present in a small proportion of PDAC, and Philip et al. (88) and Yoon et al. (89) found that KMT2C is frequently mutated in KRAS wild-type tumor samples of pancreatic ductal adenocarcinomas, providing a new promising therapeutic target for the targeted treatment of KRAS wild-type PDAC patients. Tumor mutation burden (TMB), a new biomarker, has shown its potential as a predictive biomarker for a variety of applications, including the correlation between different levels of TMB and the response of patients with various types of cancers to immune checkpoint inhibitors (ICIs) (90).

#### Colorectal cancer

KMT2C is considered an oncogene in colorectal cancer. It has been found to be frequently mutated in colorectal cancer (91) and is commonly mutated in both primary CRC and peritoneal metastases (92) and has been associated with CRC prognosis, and can be used to predict OS and PFS in patients with CRC (93, 94) It is a potential candidate for potentially identifying patients with high-risk colorectal cancer (95).

Mutations in KMT2C may be involved in the transition of non-dysplastic cells to a dysplastic phenotype in patients with long-standing ulcerative colitis (UC) and have a high risk of progression to colorectal tumors (96). Watanabe et al. found that code-shifting mutations in KMT2C in CRC cells and primary tumors were more common in cases of microsatellite instability. In addition, the CpG island-associated promoter of the KMT2C gene is not DNA methylated in CRC cells, nor is it DNA methylated in primary tumors or normal colon, and this region is highly homologous to a pseudogene for age-associated DNA methylation (psi TPTE22) (97). Meanwhile, restoration of KMT2C inhibited CRC cell growth and enhanced genome-wide histone H3 position 4-methylation deposition on enhancers; however, this effect varied depending on the histone H3 position 4-methylation status of the KMT2C-deficient cells. The results suggest that KMT2C inactivation may promote colorectal cancer development through transcriptional dysregulation of several pathways known to be associated with cancer (98). Importantly, it has been found that KMT2C loss-of-function variants (LOF) are associated with higher TMB, and specifically, KMT2C LOF variants are associated with decreased regulatory T cells and increased levels of CD8^+^T cells, activated NK cells, M1-type macrophages and M2-type macrophages in colorectal cancer (99).

### Respiratory system cancers

KMT2C mutations are very common in early stage lung adenocarcinoma and their low expression is associated with shorter overall survival time (100, 101), and the mutation frequency of KMT2C in lung adenocarcinoma patients was shown to be significantly higher in smokers than in non-smokers (102), and more importantly, variants in the gene were associated with younger lung adenocarcinoma patients (103).

Distant organ metastases are still the leading cause of cancer-related deaths (104), and the KMT2C gene, a high-frequency mutated gene common to both primary and metastatic foci of lung cancer, may provide a potential biomarker for immune checkpoint blockade in LUAD with metastases to different organs (105).

For lung cancer, the most common sites of metastasis are the contralateral lung, brain, bone, and liver (106, 107), and the study of Gao et al. (104) found that in the whole cohort of primary (PR) and metastatic foci (MT - liver, MT - bone, and MT - brain), the frequency of mutations in the KMT2C gene was high and the mutations were common to PR and metastatic foci (MT - liver, MT - bone, and MT - brain), and in the MT - bone, LRP1B mutations co-occurred with KMT2C mutations. Meanwhile, Liu et al. found that KMT2C mutations were associated with lung cancer metastasis to the brain by whole exome sequencing (108).

The development of specific antibodies against the programmed death (PD1) receptor, its ligand PD-L1 (programmed death ligand-1), and the cytotoxic T-lymphocyte-associated protein 4 (CTLA-4) receptor in first- or second-line therapeutic strategies for patients with non-small-cell lung cancer has led to an unprecedented prolongation of patient survival in the last decade (109). Therefore, there is an urgent need for effective biomarkers to predict the response of non-small cell lung cancer (NSCLC), especially NSCLC with low tumor mutation load, to immune checkpoint block (ICB) therapy (110). Gu et al. found that KRAS and KMT2C co-mutations improved the response to immunotherapy in patients with non-small cell lung cancer (111). The study by Bai et al. highlighted the potential predictive value of KMT2C for immunotherapeutic benefit in non-squamous NSCLC, and furthermore, the combination of KMT2C and PD-L1 could serve as the best partner for guiding therapeutic decisions based on anti-PD-(L)1 (112). In a recent study, researchers found that mutations in three chromatin remodeling-associated genes, KMT2C, BCOR and KDM5C, were associated with ICB responses in NSCLC, including NSCLC with low TMB levels. Moreover, the combination of KMT2C mutation with TMB or PD - L1 expression further improved this correlation. These data suggest the potential of KMT2C mutation status as a predictive biomarker for ICB therapy in NSCLC. KMT2C mutations were shown to have potential as predictive biomarkers for ICB therapy in NSCLC alone or in combination with PD - L1 expression or TMB (110).

### Urogenital system cancers

#### Bladder cancer

KMT2C plays a repressive role in bladder cancer and is one of the most commonly mutated genes in BCa patients (113). Specifically, downregulation of KMT2C in bladder cancer cells results in widespread changes in epigenetic status and expression of DNA damage response and DNA repair genes. More specifically, cells with low KMT2C activity lack homologous recombination-mediated DNA repair of double-strand breaks, and as a result, these cells suffer much higher levels of endogenous DNA damage and genomic instability. Finally, these cells appear to be heavily dependent on PARP1/2 for DNA repair and treatment with the PARP1/2 inhibitor Olaparib results in synthetic lethality, suggesting that low KMT2C-expressing cancer cells are an attractive target for PARP1/2 inhibitor therapy (61).

#### Prostate cancer

KMT2C has been described as the most mutated epigenetic regulator and driver in PCa tumor tissues (114).Coelho et al. suggest that the KMT2C gene may play as important a role in tumor suppression in PCa patients as BRCA2 (115). Moreover, alterations in KMT2C are more likely to coincide with alterations in TP53, suggesting a more aggressive phenotype in PCa, which correlates with sensitivity to treatment with poly ADT-ribose polymerase (PARP) inhibitors (116). KMT2C mutations indicate rapid progression during conventional combined antiandrogen blockade (CAB) therapy and may serve as a potential biomarker for predicting response to prostate cancer therapy (117).

Currently, high-risk human papillomavirus (hr HPV) infection is also considered a risk factor for PCa (118), and KMT2C, KMT2D and ERCC2 mutations are more frequent in HPV-positive tumors (119). The frequency of mutations in PREX2, PTEN, AGO2, and KMT2C was significantly higher in patients with a history of smoking than in nonsmokers. Smokers (p = 0.006) had a significantly higher overall mortality rate (28.5% versus 22.8%) (120). In a recent study, the authors found that KMT2C mutations were associated with PCa metastasis, and in addition, they found that KMT2C mutations were associated with reduced PCa disease-free survival, and that inhibition of the MYC signaling axis may be a viable therapeutic option for patients with KMT2C truncation (121).

#### Breast cancer

KMT2C is a gene with a high number of mutations found in breast cancer, some of which are associated with chromatin function, affecting transcriptional mechanisms identified in breast tumorigenesis and development (122) and, in addition, KMT2C mutations observed in circulating tumor DNA (ctDNA) from six-month postoperative samples may be indicative of breast cancer recurrence and prognosis (123). Alterations in KMT2C are significantly enriched in metastatic populations compared to primary breast cancers (124). KMT2C is mutated at a higher rate in HR +/HER2 type breast cancers than in other subtypes (59). KMT2C mutations have also been shown to be critical for ER α regulation, which can lead to hormone-driven proliferation of breast cancer cells (125).

Role of KMT2C as a key chromatin regulatory protein in enhancer elements and as a factor that promotes histone H3 position 4-methylation deposition in these enhancers. The precursor factor FOXA1 interacts with the chromatin-modifying factor KMT2C to promote monomethylation of H3K4 on enhancer elements, thereby generating the potential for transcription from these enhancer regions (35). In the presence of E2, KMT2C and KMT2D, together with ER, regulate the expression of the HOXC10 gene and promote breast cancer progression (126).

#### Ovarian cancer

Huang et al. found that overexpression of XIST enhanced the anticancer sensitivity of paclitaxel on ovarian cancer cells, and its effect may be related to the up-regulation of KMT2C. XIST affects the expression of KMT2C in ovarian cancer by enhancing the stability of KMT2C mRNA through the direct targeting of mi -93-5p. The results of this study suggest that the miR-93-5p/XIST/KMT2C signaling axis may provide new potential therapeutic targets for ovarian cancer treatment and play an important role in future ovarian cancer therapy (127).

### Blood system cancers

KMT2C is localized at 7q36 and was first described as a chromosomal region frequently missing in myeloid leukemia (128).

KMT2C levels decrease during progression of chronic granulocytic leukemia and correlate with different clinical stages. After treatment of the imatinib mesylate (IM)-sensitive CML cell line KCL22S with Dasatinib or Nilotinib, a restoration of KMT2C gene expression and a higher rate of apoptosis and enhanced expression of p21 (CDKN1A) compared to the control group was observed, accompanied by a decrease in the expression of CDK2, CDK4, and Cyclin B1 (CCNB1), which suggests that the p53 regulatory pathway is involved in the regulation of cancer by KMT2C (49).

### Nervous system cancers

KMT2C was one of the first few recurrently mutated genes identified in a sequencing study of early medulloblastoma (129). In the adult medulloblastoma cohort in the Jones et al. study, KMT2C was one of the most commonly mutated genes, with 30% of mutations detected in cases of different ages, sexes, histological types, and molecular typologies, again demonstrating the central importance of chromatin modifications in the pathophysiology of medulloblastomas (130) and highlighting the fact that a more comprehensive review of the adult medulloblastoma epigenetic landscape is the need for a more comprehensive assessment (131). In another study, researchers identified inactivating mutations in the histone lysine N -methyltransferase genes KMT2C or KMT2D in 16% of patients with childhood medulloblastoma (MB) (129).

Alterations in KMT2C at the gene level or at the protein level may disrupt the epigenetic programme and lead to malignant transformation of gliomas (132).Kleefstra et al. (133) found that an autosomal dominant nonsense mutation (p. Arg1481 *) in KMT2C leads to neurodevelopmental disorders manifested by mental retardation, growth retardation, mild dysmorphic features (including prominent eyebrows, thick ear whorls, and misaligned teeth), and neuropsychiatric traits, including hyperactivity and aggression. Mutations in KMT2C in neurodevelopmental disorders have also been described by Vallianatos et al. (134).

## Future prospects and conclusions

Post-translational modifications are typical biochemical reactions that covalently bind (poly)peptide chains, chemical parts, lipids or carbohydrates to amino acids of a target molecule during or after translation. PTMs occur in most known proteins, and virtually all amino acids can be changed by one or more of these reactions. Modified proteins gain uncommon amino acids that can have a significant impact on their structure and function. Post-translational modifications diversify the proteome by altering the structure, location, interactions, and function of proteins and their regulation, thereby affecting various functional aspects of the cell. Methylation is an important post-translational modification that regulates various biological functions of cells by modifying proteins. In recent years, due to increased research on tumor progression and treatment, more and more researchers have begun to focus on methylation in the search for effective anti-tumor therapy.

KMT2C is the writer of the methylation process that is widespread in eukaryotes. it plays an essential role in histone methylation modification. KMT2C is frequently mutated in a variety of human cancers, including hepatocellular carcinoma, non-small-cell lung cancer, and breast cancer. KMT2C stimulates the development, progression and metastasis of human tumors by regulating different signaling pathways associated with human tumors, as well as a variety of proteins that are not involved in the above signaling pathways. The EMT signaling pathway promotes tumor cell migration and invasion, and is an important tumor-promoting factor. KMT2C inhibits the expression of proteins associated with the EMT signaling pathway, which may provide a direction for the development of new anticancer drugs. However, the regulation of GSK3b/p65, DSB repair and other signaling pathways by KMT2C needs to be further investigated, and the specific mechanism of KMT2C’s action in other tumors and its potential as a novel anti-tumor therapy need to be further studied. Although previous findings have shown that KMT2C is a promising target for the treatment of cervical and bladder cancers, the specific molecular mechanisms involved in the regulation of KMT2C in cervical and bladder cancers remain to be further investigated. We suggest that future studies focus on the pathways that promote cancer progression activated by KMT2C mutations, develop inhibitors of the corresponding pathways and demonstrate their efficacy and safety in treating these diseases.

## Author contributions

YJ: Writing – original draft. YHL: Writing – original draft. ML: Writing – review & editing. YL: Writing – review & editing. MH: Writing – review & editing. XX: Writing – review & editing. HZ: Writing – review & editing. JZ: Conceptualization, Writing – review & editing. XK: Conceptualization, Writing – review & editing. WS: Conceptualization, Writing – review & editing.
